# Intimacy Effects on Action Regulation: Retrieval of Observationally Acquired Stimulus–Response Bindings in Romantically Involved Interaction Partners Versus Strangers

**DOI:** 10.3389/fpsyg.2018.01369

**Published:** 2018-08-03

**Authors:** Carina Giesen, Virginia Löhl, Klaus Rothermund, Nicolas Koranyi

**Affiliations:** General Psychology II, Institute of Psychology, Friedrich Schiller University Jena, Jena, Germany

**Keywords:** stimulus–response binding, event files, joint action, romantic relationship, observational learning

## Abstract

Previous research has shown that stimulus–response (SR) binding and retrieval processes also occur when responses are only observed in another person (Giesen et al., 2014). Importantly, this effect depends on the two individuals interacting interdependently during the task (e.g., competition or cooperation). Interdependence, however, must not necessarily result from task-related demands, but can also reflect an intrinsic feature of a given relationship. The present study examines whether observing responses of one’s romantic partner also produces stimulus-based retrieval of observed responses even if the task itself does not involve interdependence. Participants performed a task pairwise, either with their romantic partner or with a stranger. In a sequential prime-probe design, both participants of a pair gave color responses themselves (actors) or merely observed these (observers) in alternating fashion. As expected, stimulus-based retrieval of observationally acquired SR-bindings occurred only in romantically involved pairs; participants interacting with a stranger showed no retrieval effects. We conclude that mental representations of self and other are more closely intertwined in romantic couples, which produces automatic retrieval of observationally acquired SR binding effects even independently of the task itself.

## Introduction

“Only let me assure you, my dear Miss Elizabeth, that I can from my heart most cordially wish you equal felicity in marriage. My dear Charlotte and I have but one mind and one way of thinking. There is in everything a most remarkable resemblance of character and ideas between us.”

Mr Collins, *Pride and Prejudice* by Jane Austen

It is an obvious truth that relationships with other people represent a central aspect of our social lives and influence our thinking, feeling, and behavior in various ways. Among the numerous relationships that we initiate and maintain, the one that we have to our romantic partner or spouse is a special one. The relationship partner is of primary significance for satisfying fundamental affiliation and intimacy motives (Baumeister and Leary, 1995) and it is in most cases him/her to whom we turn to when we need someone to talk to or when support is needed in stressful times (e.g., Coyne and DeLongis, 1986; Revenson, 1994).

A core characteristic of satisfied and stable couples is that the relationship partners display high interdependence in thoughts and feelings and strongly co-ordinate their behavior (Agnew et al., 1998). Specifically, it has been shown that high-functioning couples have a strong tendency to match their responses to tasks or challenges and thereby become rather effective in dealing with everyday stress and developmental tasks (e.g., Bodenmann et al., 2006; Papp and Witt, 2010; Neff and Broady, 2011).

To date, joint action regulation in intimate relationships has typically been examined on the macro-level, for instance by assessing couple’s overt responses to a (demanding) task either by observational procedures or self-reports. In contrast, the underlying cognitive micro-processes of joint action regulation have received far less attention. The present research tries to fill this gap by combining the “couple perspective” with recent advances in research on social influences in automatic joint action regulation. Specifically, the present study focuses on stimulus–response (SR) binding and retrieval processes which reflect a fundamental mechanism of action automatization. It will be argued that due to the high relevance of one’s relationship partner, SR binding and retrieval also occurs by mere observation of one’s romantic partner and thereby forms the basis for dyadic behavior coordination on a more elaborate level.

### Stimulus–Response (SR) Binding and Retrieval Processes

Processes of stimulus–response (SR) binding and retrieval depict a fundamental process of automatic action regulation (Logan, 1988): That is, whenever a response is made to a stimulus in a given (*prime*) trial, the mental representations of stimulus and response will be transiently bound together in an SR binding or *event file* (Hommel, 1998). Repeating one element of this binding in a subsequent (*probe*) trial (e.g., a stimulus repetition probe), will retrieve the entire SR binding from memory, meaning that re-execution of the previous response is facilitated. If the retrieved prime response is also appropriate in the current probe trial, SR retrieval will produce performance benefits, compared with a situation without stimulus repetition (i.e., a stimulus change probe). However, if the retrieved response is inappropriate in the current probe trial, SR retrieval will produce performance costs (relative to stimulus change probes, respectively; Rothermund et al., 2005). To date, a burgeoning amount of evidence documents that processes of SR binding and retrieval apply to a broad scope of stimuli, modalities, and responses (see Henson et al., 2014, for an overview), and thus play a dominant role for automatic action regulation.

**FIGURE 1:**
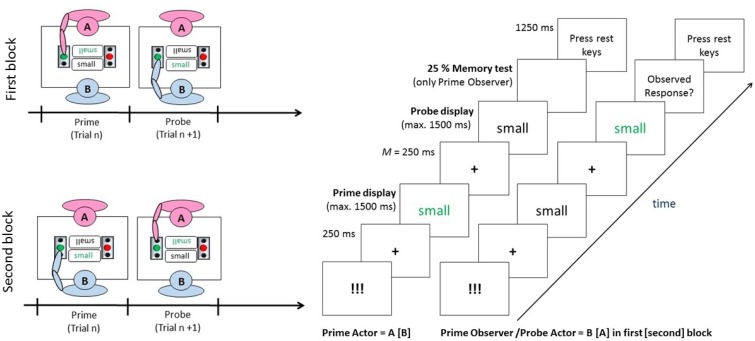
Schematic overview of the experimental setup for participants A and B (left side) and exemplary prime-probe sequence from each participant’s perspective (right side). In the first block, participant A was prime actor and participant B was prime observer/probe actor. In the second block, this assignment was reversed, meaning that participant B was prime actor and participant A was prime observer/probe actor. Pairs were always opposite-sex interaction partners who were either romantically involved with each other (“romantic partner” condition) or were both in a romantic relationship with someone else (“stranger” condition). Assignment of males (blue figures)/females (pink figures) to the roles of prime actor vs. observer was random. Stimuli are not drawn to scale.

Since the seminal work by Albert Bandura on social learning by observation, it is known that most of our action routines are not based on our own experience, but result from the observation of others. However, one will not blindly copy any action observed in another person. On the contrary, particular moderating conditions determine to which extend one will incorporate an observed response in one’s own action repertoire (Bandura, 1986). Intriguingly, principles of observational learning may also (and to a similar extend) influence micro-processes of automatic action regulation. For instance, recent studies revealed that response execution is no necessary pre-condition for the formation of SR bindings: Notably, SR bindings are also created if the response to a stimulus is only *observed* in another person (Giesen et al., 2014, 2017). In their study, Giesen et al. (2014) created a joint version of the standard SR binding task. Two participants performed a shared color categorization task. One participant categorized the color of a word stimulus presented in the prime trial (prime actor). At the same time, the other participant (prime observer) only saw the word, but no color, and had to observe the prime response that was given by the prime actor – which should lead to the formation of an observational SR binding. Crucially, to test whether SR bindings were indeed acquired by observation, the former prime observer became probe actor and had to categorize the color of a word stimulus presented during the probe trial (see **Figure 1**). Stimulus relation from prime to probe (i.e., word repetition versus word change) and compatibility between observed prime responses and to-be-performed probe response (i.e., compatible vs. incompatible) were manipulated orthogonally. Analogously to the logic of “standard” SR retrieval effects, probe trials with stimulus repetition should trigger retrieval of the observationally acquired SR binding. The crucial question was thus whether probe actors’ performance in the probe would reflect a pattern that is consistent with SR retrieval effects (indicated by a Stimulus Relation × Response Compatibility interaction). Indeed, this was the case: When to-be-performed probe responses were compatible with observed prime responses, performance was faster on probe trials with stimulus repetition than on stimulus change probes (yielding performance benefits due to SR retrieval of “appropriate” responses). However, when to-be-performed probe responses were incompatible with observed prime responses, performance was slower on stimulus repetition probes than on stimulus change probes (yielding performance costs due to SR retrieval of “inappropriate” interfering responses).

Crucially (and in analogous fashion to social learning phenomena on the macro level, see Bandura, 1986) social dependence among pairs of interacting participants during the task modulated this pattern of results. Giesen et al. (2014) contrasted three conditions: Some pairs of participants had to *cooperate* to gain an extra reward (a chocolate bar): pairs were informed that both participants would gain the extra reward if – and only if – both performed well in terms of response speed and accuracy; otherwise, both would get no extra reward. In a second group, pairs had to *compete against* each other, meaning that only the better participant of each pair would gain the extra reward, whereas the other would leave empty handed. In the last group, participants worked *independently* of each other to gain the extra reward, meaning that distribution of the reward depended solely on participants’ individual performance. This manipulation of social interdependency between co-actors had a considerable influence on retrieval of observationally acquired SR bindings: only participants who were socially dependent on their co-actor (i.e., pairs in the cooperative or competitive condition) showed retrieval of observational SR bindings. In turn, participants who did not depend on their co-actor to gain the extra reward showed no retrieval effects at all. These findings attest that retrieval of observationally acquired SR bindings is a conditionally automatic process that is contingent on the situational interdependency between interaction partners.

The idea that observed actions are mentally represented like one’s own actions is central for a range of paradigms that investigate related phenomena like observational acquisition of action-effect bindings (Paulus et al., 2011), imitation tasks (e.g., Brass et al., 2001; van Baaren et al., 2009), or co-representation effects eminent in interactive/joint action tasks like the Joint Simon task (Sebanz et al., 2003). It is noteworthy that the type of social relation during the task is a strong modulating influence in these paradigms as well. For instance, interference effects in the Joint Simon task are also stronger as social relations become more interdependent (Hommel et al., 2009; Ruys and Aarts, 2010; Iani et al., 2011; Müller et al., 2011); the same holds true for effects of unconscious imitation (mimicry; van Baaren et al., 2009).

In previous research on stimulus-based retrieval of observed responses, interdependence between two individuals was situationally induced by instructing participants to cooperate with or compete against each other (Giesen et al., 2014). Interdependence, however, must not necessarily be the result of task-related demands, but can also reflect a permanent feature of a given relationship. Romantic relationships reflect a paramount example in this respect (Aron et al., 1991). According to Aron et al. (1991, p. 242), the interdependent structure of romantic relationship even implies that “the person acts as if some or all aspects of the partner are partially the person’s own.” Thus, persons tend to represent their romantic partner in their mental “self” representations to a considerable extent (this aspect is nicely illustrated in the starting quote). Furthermore, romantic partners perceive themselves less individualistic and more as part of a “self-and-partner” collective (Agnew et al., 1998).

### Aims of the Present Study

In the present study, we examined whether romantic relationships exert an influence on the retrieval of observational SR bindings that mimics the effects of social dependence documented by Giesen et al. (2014). To this end, we only recruited participants who were involved in a committed relationship. Participants first answered an online questionnaire in which we assessed relationship quality (among other measures). Then, pairs of two participants were invited to the lab, consisting either of the two partners of a relationship or of two people from different relationships. Participants thus worked through the observational SR binding task either with their romantic partner or with a stranger. Note that relationship status was constant between groups. In other words, groups only differed in whether pairs of participants were romantically involved with *each other* (“romantic partner” condition) or with *someone else* (“stranger” condition). We expected that working with one’s own romantic partner (compared with working with a stranger) should directly influence retrieval of observational SR bindings as a function of attention. Specifically, participants are likely to regard actions performed by their romantic partner as more relevant and consequently attend more to them. According to Logan (1988), attention is not only beneficial for encoding, but also for retrieving SR episodes (see also Moeller and Frings, 2014). Furthermore, we reasoned that this should hold true not only for “standard” SR episodes (i.e., transient bindings between stimuli and self-performed responses), but also for bindings of stimuli and observed responses. Thus, if attention due to increased relevance of actions performed by one’s romantic partner (vs. a stranger) is critical for the retrieval of observationally acquired SR bindings, the Interaction Partner × Stimulus Relation × Response Compatibility three-way interaction should be significant. Specifically, we expected that (a) probe actors’ performance of participants in the “romantic partners” condition reflect a pattern that is indicative of SR retrieval. In statistical terms, retrieval of observationally acquired SR bindings is indicated by an *interaction* of the factors stimulus relation (repetition vs. change) and response compatibility between observed prime and to-be-performed probe responses (compatible vs. incompatible). In other words, stimulus repetition in the probe trial should retrieve observationally acquired SR bindings from memory, reactivating the observed prime response. Thus, when to-be-performed probe responses are compatible with observed prime responses, performance should be faster on stimulus repetition probes, compared with stimulus change probes. In turn, when to-be-performed probe responses are incompatible with observed prime responses, performance should be slower on stimulus repetition probes, compared to stimulus change probes. Furthermore, based on the findings of Giesen et al. (2014), we expected that (b) SR retrieval effects should be absent for probe actors in the “stranger” condition (i.e., no Stimulus Relation × Response Compatibility interaction), because the task itself did not create any kind of interdependence between the participants of the pair.

## Materials and Methods

### Participants

According to *a priori* calculations with the G^∗^Power 3.1 software (Faul et al., 2007), a sample size of *n* = 27 per “interaction partner” condition is required to guarantee sufficient statistical power of 1-β = 0.80 with α = 0.05 to detect a medium-sized (*d* = 0.50) effect in the “romantic partners” condition (where we predicted to find an effect that is statistically different from zero) and in the “strangers condition” (for which we predicted a null-effect).

The study took place in a predetermined time period during which the lab was available. Recruiting of romantic couples of which both participants could take part turned out to be particularly challenging. In the given time period, we managed to recruit 52 native German-speaking participants for the experiment (32 female^1^; age*: M* = 24.9 years, *SD* = 7.1; relationship duration: *M* = 3.4 years, *SD* = 4.1). Participants were either students at FSU Jena (*n* = 40), received other educational training (*n* = 4), or were already working (*n* = 8). All participants were involved in a permanent, committed heterosexual romantic relationship. Due to an error in recruitment lists, resulting sample size per condition was slightly off-balanced (*n* = 22 for the “romantic partners” condition; *n* = 30 to the “stranger” condition). Since the recruited sample sizes deviated from those calculated in *a priori* power analyses, we performed *post hoc* power calculations with G^∗^Power to check the achieved power of each condition. Calculations showed that achieved power to detect a medium-sized effect (*d* = 0.50) was 1-β > 0.73 in the “romantic partners” condition (meaning that this condition was slightly under-powered) and 1-β > 0.84 in the stranger condition (meaning that this condition was sufficiently powered, which is especially important since we predicted a null finding).

Ethical approval of the study was granted by the Ethical Commission of the Faculty of Social and Behavioural Sciences, FSU Jena (FSV 18/25). All participants provided written informed consent.

All participants answered a brief online questionnaire (5 min) individually at home. In the lab, participants performed the computer experiment in pairs and then answered another brief questionnaire on their own. Lab sessions lasted 45–50 min. Participants received partial course credits or sweets for their voluntary participation. To incentivize participation, three *Amazon* vouchers (£15; £10; £5) were raffled among all participants. If participants showed an appropriate performance during the computer experiment, participants received more sweets as an extra reward. Importantly, distribution of the extra reward depended solely on the participants’ individual performance and not on their partners’ performance (“independence” condition of Giesen et al., 2014).

### Experimental Set-Up and Stimuli

During the computer experiment, two participants sat opposite to each other at a table, each one in front of a 19-in. flat-screen monitor to prevent participants’ direct eye contact. The experiment was programmed with E-Prime 2.0. On each participant’s monitor, word stimuli (25 neutral, frequently used German adjectives that were either mono- or disyllabic and consisted of four to seven letters) were presented in Times New Roman 16-pt font centrally on a blank black screen. Two response pads – one with a red and one with a green push-button in the middle and two black rest-state keys in front of and behind each push-buttons (see **Figure 1**) – were fastened to the table and served to collect responses. In detail, participants permanently pressed the rest-state keys with their left and right hand, respectively. Each participant had the task to categorize the color of the presented word stimulus. Participants performed this task in turns (i.e., only one participant saw a colored word stimulus, whereas the other saw the word stimuli presented in white font; see **Figure 1**). They gave their responses by releasing one of the rest-state keys to hit the according (red or green) push-button in front of the released rest-state key. The response pads were connected to the computer via the parallel port to collect the color categorization responses. Both the release response of the rest-state keys and the hit responses of the red/green push-buttons were measured, but only the release response reaction times (RTs) was used for analysis. That is because probe hit responses are confounded with movement speed (i.e., time to reach the push buttons). Release RT represent a more pure measure of the time it took participants to initiate a response.

### Procedure

The current study consisted of three different parts. First, an online questionnaire requested personal demographics. To prove the inclusion criterion, participants had to report their relationship status, relationship duration, and sexual orientation. Furthermore, we assessed general relationship satisfaction with the German version of the “Relationship Assessment Scale” (RAS; Sander and Böcker, 1993). Participants answered seven items containing questions about their current romantic relationship. Using 5-point scales, they were asked to rate their relationship as 1 (*low satisfaction*) versus 5 (*high satisfaction*). Items 4 and 7 of the scale are reverse coded (Cronbach’s alpha = 0.75). Second, after answering the online questionnaire at home, participants were invited to the lab to take part in the computer experiment. Two participants (referred to as Participants A and B) worked in a pair and performed a color categorization task in alternating fashion (see Giesen et al., 2014, Experiment 1, for a similar procedure). For both participants, instructions were presented on each participant’s screen. Participants were able to determine the duration of reading the instructions individually. For both prime and probe displays, participants’ task was to categorize the color of the presented word stimuli by pressing the corresponding (i.e., red/green) push-button in the middle of the response pads. Thus, color of word stimuli was task-relevant, whereas the identity/meaning of the word was irrelevant in prime and probe displays and served as a distractor (Rothermund et al., 2005). Importantly, the color categorization task was shared between both participants. Hence, only one participant of each pair saw a colored word during the prime or probe display (the actor). For the other participant (the observer) the same word was presented in white font (see **Figure 1**). In particular, for the first 160 prime-probe sequences, Participant A was “prime actor” and had to categorize the color of word stimuli presented during the prime display. By implication, participant B was “prime observer” and had the task to observe the color categorization response carried out by the prime actor. Importantly, participant B then became “probe actor” and had to categorize the color of the word stimulus presented during the probe display. By implication, participant A was “probe observer” and had to observe the color categorization response carried out by the probe actor. For the remaining 160 prime-probe sequences, participant B was the prime actor/probe observer and participant A the prime observer/probe actor. This was done to collect probe responses from both participants, since we used probe actors’ release RTs as primary dependent variable for the analyses of interest.

Each prime-probe sequence followed the pattern shown in **Figure 1** (right side). First, as a ready signal, three exclamation marks were displayed centrally in each participant’s screen in white font for 500 ms. After that, a fixation cross appeared for 250 ms. Subsequently, the prime display started with a word stimulus presented in red or green font for the prime actor and in white font for the prime observer. Stimuli remained on screen until prime actors hit one of the push-buttons to categorize the word color or until a maximal duration of 1,500 ms had elapsed. Immediately after the prime actor resumed to press both rest-state keys, another fixation cross appeared for a duration that varied randomly between 150 and 350 ms (*M* = 250 ms). The duration was variable between sequences to prevent an exact anticipation of the probe display’s onset. Then the probe display started with another word stimulus presented in red or green font for the probe actor and in white font for the probe observer. Stimuli remained on screen until probe actors hit one of the push-buttons to categorize the word color or until a maximal duration of 1,500 ms had elapsed. Immediately after the probe actor resumed pressing both rest-state keys, the experiment continued as follows. In 25% of randomly selected prime-probe sequences, a memory test for the prime observer appeared after the probe display. The memory test served to ensure that prime observers attended to color responses of prime actors. Prime observers had to press the push-button that corresponded to the observed (prime) response. The memory test remained on screen until one of the push-buttons was pressed. Once prime observers continued pressing both rest-state keys, a black screen appeared for 1,250 ms, reminding participants to keep both rest-state keys pressed. Then, the next prime-probe sequence started.

Participants performed a practice block of 32 prime-probe sequences before starting the first experimental block. Only the practice block included immediate feedback for erroneous or too slow responses. If release responses were slower than 750 ms, the message “Respond faster!” was displayed. If actors in prime and probe hit the wrong push-button, the message “Error–wrong key!” appeared. If the wrong person released a rest-state key, the message “Error–wrong person!” appeared. All feedback messages were shown to both participants centrally on a red background in white font for 1,000 ms. If participants performed too many erroneous or too slow responses in the practice block, a second practice block followed. Upon successful completion of the practice, participants were informed that they worked independently of their interaction partner (Giesen et al., 2014), meaning that distribution of the extra reward for each of the two participants depended only on their own individual performance. Participants then worked through two experimental blocks comprising of 160 prime-probe sequences each. After every 40 prime-probe sequences, both interaction partners received a short feedback on their own performance (% errors; % slow responses).

Third, after completion of the computer task, participants received a brief paper questionnaire to assess how participants perceived the situation and their interaction partner during the task. Using 7-point bipolar scales, three items assessed participants’ experienced discomfort versus comfort during the experiment (i.e., 1 = *difficult/unpleasant/negative*; vs. 7 = *easy/pleasant/positive* Cronbach’s alpha = 0.73). Additionally, participants were asked to rate the experimental situation as 1 (*competitive*) versus 7 (*cooperative*). With four other items participants were further asked to indicate the impression the interaction partner had left (i.e., 1 = *disagreeable/insecure/unfriendly/incompetent* vs. 7 = *agreeable/confident/friendly/competent*; Cronbach’s alpha = 0.94). Using a 5-point scale, participants were asked whether they were acquainted with their interaction partner (1 = not at all vs. 5 = very familiar). A last dichotomous item asked whether they had used any strategies to perform the task. After completion of the questionnaire, participants were thanked and rewarded. Participants received the extra reward if more than 75% responses were faster than 750 ms, if less than 10% of color categorizations and less than 20% of memory tests were erroneous. Further, participants could deposit their own e-mail address to receive a debriefing.

### Design

The experimental design comprised a 2 × 2 × 2 mixed-factors design with the within-subject factors stimulus relation and response compatibility and the between-subject factor interaction partner. Stimulus relation was manipulated by presenting the same prime word in the probe in 50% of all prime-probe sequences (word repetition, e.g., small–small) and by presenting a probe word differing from the previously presented prime word in 50% of all prime-probe sequences (stimulus change/baseline, e.g., quiet–small). Response compatibility was varied by requiring probe responses that were compatible to observed prime responses in 50% of all prime-probe-sequences (compatible response, e.g., red–red) and by requiring probe responses that were incompatible to observed prime responses in 50% of all prime-probe sequences (incompatible response, e.g., green–red). The between factor (interaction partner) was manipulated by assigning participants either to work with their romantic partner (*n* = 22) or to perform the task together with a stranger (*n* = 30). Condition assignment depended partially on how feasible it was for participants to bring their romantic partner to the lab. To achieve homogenous and comparable groups, all participants were involved in heterosexual romantic relationships and worked with an opposite-sex interaction partner during the experimental session. Details on relationship ratings in both conditions are reported below. Release reaction time (RT) of the rest-state keys in the probe served as the primary dependent variable during the color categorization task. However, analyses of probe hit RTs yielded very similar results (see Footnote 3). Since probe hit RT are confounded with movement speed, we refrained from interpreting any results relating to probe hit RTs.

Font color of prime words was counterbalanced (50% of all prime stimuli were presented in red, 50% were presented in green to the prime actor). Likewise, font color of probe words was counterbalanced (50% red; 50% green; note that probe color depended on the experimental factor response compatibility).

## Results

All statistical analyses were performed with R.

### Manipulation Checks

#### Ratings of Experimental Situation and Interaction Partner

We computed mean ratings of participants’ perception of the experimental situation and their interaction partner for both interaction partner conditions (see **Table 1**). Results indicated that the interaction conditions differed significantly only with respect to the perceived (dis)agreeableness of the interaction partner. Not surprisingly, romantically involved interaction partners judged each other as more agreeable, confident, friendly, and competent (*M* = 6.1, *SD* = 1.1) than interaction partners in the “stranger” condition (*M* = 4.8, *SD* = 1.3), *t*(50) = 2.99, *p* = 0.004. The interaction partner conditions did not differ significantly with respect to ratings of perceived (dis)comfort of the situation, *t*(50) = 1.84, *p* = 0.07, and to the question how cooperative/competitive they experienced the situation, *t*(50) = 1.58, *p* = 0.12. Cooperation/competition and perception of the situation thus seem to be unaffected by the interaction partner manipulation. Participants in the stranger condition reported not to be acquainted with their interaction partner (*M* = 1.4, *SD* = 0.7). Naturally, romantic partners were acquainted with each other (*M* = 5.0, *SD* = 0.0). Acquaintance scores differed significantly between both conditions, *t*(50) = -23.66, *p* < 0.001.

**Table 1:** Means (*SD*) of participants’ ratings of the experimental situation and memory test performance.

	Interaction partner	
	Stranger *n* = 30	Romantic partner *n* = 22
**Situation perceived as**		
Comfortable (7) vs. uncomfortable (1)	4.9_a_ (1.2)	5.5_a_ (1.1)
Cooperative (7) vs. competitive (1)	4.8_a_ (1.6)	5.5_a_ (1.3)
**Interaction partner perceived as**		
Agreeable (7) vs. disagreeable (1)	5.1_a_ (1.3)	6.1_b_ (1.1)
Memory test performance (error rate)	0.03_a_ (0.06)	0.03_a_ (0.03)

*Means in the same row with different subscripts differed at p < 0.01.*

#### Relationship Ratings

As part of the online questionnaire, participants rated their relationship satisfaction with the RAS before taking part in the computer experiment. We computed the average RAS scores (cf. Sander and Böcker, 1993) separately for each participant. In general, RAS scores were rather high. Importantly, however, the relationship satisfaction of participants who interacted with their romantic partner (*M* = 4.3, *SD* = 0.4) did not differ from the relationship satisfaction of participants who interacted with a stranger (*M* = 4.3, *SD* = 0.4), |*t*| < 1. However, and unexpectedly, the duration of the current relationship differed significantly between both interaction groups: Relationship duration was longer for participants in the “romantic interaction partners” condition (*M* = 4.8 years, *SD* = 5.7 years) than for participants in the “stranger” condition (*M* = 2.3 years, *SD* = 1.7 years), *t*(50) = -2.21, *p* = 0.032. *Post hoc* data exploration revealed that this difference was due to two outliers in the romantic partner sub-sample (i.e., a couple with very long relationship duration). When this outlier couple was removed, relationship duration no longer differed between both interaction partner conditions^2^ .

#### Memory Test Performance

Additionally, we computed probe actors’ average error rates in the memory test (see **Table 1**) to ensure that participants of both conditions were motivated to a comparable extent to observe their interaction partner’s prime reactions. Error rates were low in general (3.0%); most importantly, they did not differ between interaction partner conditions, |*t*| < 1. We conclude that all prime observers adequately attended and thus memorized their interaction partner’s prime response.

### Probe Performance

Only probe actors’ release RTs after correct prime responses and for correct probe responses were analyzed. Thus, 1.6% prime-probe sequences with erroneous responses of the prime and/or probe actor were excluded. We also excluded probe responses for sequences with erroneous responses in the memory test (3.0%; overall: 0.7%) and probe release RT outlier values^3^ (5.4%). We then computed probe actors’ mean release RTs for every condition of the factorial design (see **Table 2**). These means were entered into a 2 (stimulus relation: stimulus repetition vs. stimulus change/baseline) × 2 (response compatibility: compatible vs. incompatible) × 2 (interaction partner: romantic partners vs. strangers) mixed factor analysis of variance (ANOVA).^4^

**Table 2:** Means (*SD*) of probe actors’ release RT (ms).

		Stimulus relation			
Interaction partner	Response compatibility (R)	SR	SC	SR-effect (=SC - SR)	S × R interaction effect [=(SC - SR)_C_ - (SC - SR)_IC_]
Stranger *n* = 30	C	434 (51)	438 (51)	4 [3.2]	1 [4.7]
	IC	445 (46)	448 (45)	3 [2.5]	
					
Romantic partner *n* = 22	C	475 (84)	484 (92)	9 [4.0]	16 [6.0]
	IC	495 (88)	488 (79)	-7 [4.4]	

*SR, stimulus repetition; SC, stimulus change (baseline); SR-effect, stimulus repetition effect, computed as difference between SC minus SR; S × R Interaction Effect, interaction between stimulus relation and response compatibility, computed as the difference between SR-effects for compatible responses minus SR-effects for incompatible responses; C, compatible; IC, incompatible. Standard errors of the means in squared brackets.*

**FIGURE 2:**
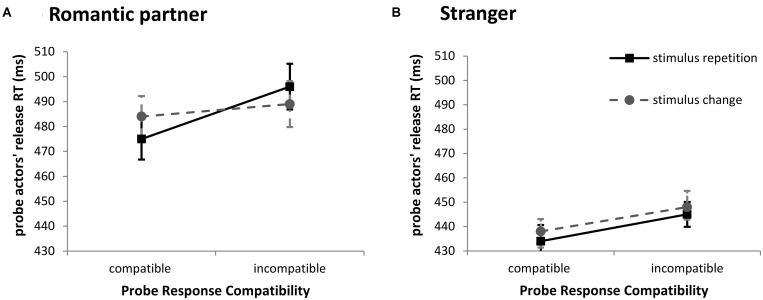
Probe actors’ average release RT (ms) as a function of stimulus relation (stimulus repetition: solid lines; stimulus change: dotted lines), response compatibility between observed prime and executed probe response, and interaction partner (**A**: probe performance of participants interacting with their own romantic partner; **B**: probe performance of participants interacting with a stranger). Error bars depict 95% confidence intervals for paired differences (CI_PD_; Pfister and Janczyk, 2013), computed for the difference of stimulus change minus stimulus repetition (SC-SR) within each probe response compatibility level.

Results revealed significant main effects of response compatibility, *F*(1,50) = 20.15, *p* < 0.001, $\eta_{p}^{2}$ = 0.29, indicating that probe actors responded faster in sequences in which a compatible probe response was required (454 ms) compared to sequences in which an incompatible response was required (466 ms). Additionally, the main effect of interaction partner was also significant, *F*(1,50) = 5.94, *p* = 0.018, $\eta_{p}^{2}$ = 0.11, showing that probe actors who worked with a stranger (441 ms) were faster than probe actors who worked with their romantic partner (486 ms) during the experiment. Most central to our prediction, the three-way interaction of stimulus relation, response compatibility, and interaction partner was significant, *F*(1,50) = 4.37, *p* = 0.042, $\eta_{p}^{2}$ = 0.08, indicating that retrieval effects for bindings between observed prime responses and word stimuli (i.e., the Stimulus Relation × Response Compatibility interaction) differed between the “romantic partner” and “strangers” condition (see **Figure 2**). To investigate the three-way interaction in more detail, we conducted follow-up ANOVAs separately for both conditions of the interaction partner factor. In line with our hypothesis, the Stimulus Relation × Response Compatibility interaction was significant in the romantic partner condition, *F*(1,21) = 7.50, *p* = 0.012, $\eta_{p}^{2}$ = 0.26 (see **Figure 2A**). When required probe responses were compatible with observed prime responses, stimulus repetition from prime to probe significantly facilitated performance compared with stimulus change probes (Δ = 9 ms; *t*[21] = 2.35, *p* = 0.014 [one-tailed], *d*_z_ = 0.50). In turn, when required probe responses were incompatible with observed prime responses, stimulus repetition from prime to probe led to a descriptive slowing of responses, compared with stimulus change probes, although this performance cost just missed conventional levels of significance (Δ = -7 ms; *t*[21] = 1.59, *p* = 0.063 [one-tailed], *d*_z_ = 0.34). In contrast, the Stimulus Relation × Response Compatibility interaction was completely absent in the stranger condition, *F* < 1, *p* = 0.868, $\eta_{p}^{2}$ = 0.00 (**Figure 2B**). No other effect was significant.

## Discussion

The present study examined stimulus-based retrieval of observationally acquired SR bindings in romantically involved couples versus pairs of strangers. We assumed that due to the interdependent structure of romantic relationships, romantically involved individuals would more closely represent their own and their partner’s actions and would do so even if the task itself does not involve interdependence (i.e., even without instruction to cooperate or compete). Consequently, retrieval of observational SR bindings should be present in romantically involved interaction partners, but should be absent in unacquainted interaction partners. The present findings support our reasoning: Stimulus-based retrieval of observationally acquired SR bindings occurred only in romantically involved pairs; prime observers interacting with a stranger showed no retrieval effects for their interaction partners’ behaviors.

Although numerically, stimulus repetition effects produced facilitation (i.e., positive) as well as interference (i.e., negative) effects for the “romantic partners” condition, the statistical pattern of stimulus repetition effects suggests that the effects are primarily driven by facilitation (i.e., significantly faster RTs in for probe trials with compatible responses), rather than interference effects (since RT differences for probe trials with incompatible responses did not differ significantly from zero). The presently observed asymmetry is not uncommon in studies on SR-binding and retrieval effects and has been reported before (e.g., Rothermund et al., 2005; Frings et al., 2007; Frings and Rothermund, 2011; Horner, 2015). However, we want to emphasize that the most central test for stimulus-based binding and retrieval effects is the interaction term (i.e., the net effect of both facilitation and interference effects); importantly, this interaction was significant for the “romantic partners” condition, but was absent (with *F* < 1) for the “stranger” condition.

Before discussing the theoretical implications of our findings, we address some alternative explanations for the present results. First, and somewhat unexpectedly, interaction partner conditions differed significantly in relationship duration. Thus, one might argue that participants in the “romantic partner” condition might have been those who are more able to enter and maintain long-lasting relationships which might be associated with a general disposition or ability to rely on observational SR retrieval. However, *post hoc* data exploration showed that this significant effect was due to an outlier couple with very long relationship duration in the “romantic partner” condition. Exclusion of this couple (a) removed any significant differences between interaction partner conditions on relationship duration, but (b) did not affect relationship quality scores between groups, which did not differ statistically. Most importantly, (c) the pattern of results obtained for probe release RT was unaffected by outlier removal since the three-way interaction remained significant (see Footnote 2 for details). We can therefore conclude that differences in retrieval of observational SR bindings between both interaction partner conditions cannot be explained by differences in relationship duration. In our view, findings are uniquely attributable to differences in mutual interdependence that accrue from interacting with a stranger or one’s romantic partner.

Second, it is possible that participants in the “romantic partner” condition implicitly assumed that their romantic partner would share her/his outcome in the experiment (as they might probably do themselves), although the distribution of extra rewards was based on each participant’s individual performance and was independent of the performance of the interaction partner. Expectation of shared outcomes is known to produce a perception of “common fate,” which is a key element of cooperative contact (e.g., Gaertner et al., 1999). This might have produced a more cooperative condition for participants in the “romantic partners” condition, compared with participants in the “stranger” condition, and thus reflects a possible alternative explanation for the observed effects^5^. However, we regard this possibility as somewhat unlikely, for several reasons: (a) Sharing outcomes might characterize only some, but not all romantic couples, and is highly influenced by various additional factors (e.g., individual preferences, personality style, etc.). We simply do not know whether and to which extent some or all of the romantic couples formed such a “common fate” perception. (b) Romantic relationships are the paramount example for positive interdependent relationships (possibly reflecting a “ceiling” effect in terms of positive interdependency). Thus, we consider it unlikely that any relationship got *even more positively interdependent than it already is* based on the mere possibility of shared profits. (c) We explicitly assessed to which extent participants perceived the experimental situation as cooperative/competitive. Importantly, both groups did not differ significantly on this measure (see “Results” section). This finding argues against any confounding influence due to expectations of “shared outcomes” or “common fate” perceptions in the “romantic partners” condition. However, we concede that most of these speculations are *post hoc*, and that it would be preferable to explicitly assess whether and to which extent the expectation of shared profits alone shaped participants perception of the experimental task as more cooperative and thus affected retrieval effects. To address this, one would need a follow-up study with the following design: Pairs of participants work independently of each other on the observational SR binding task. Importantly, participants may acquire a claim for an extra reward (based on their individual performance). However, each extra reward this then submitted to a “pool of shared profits,” which may hold none, one, or two extra rewards (based on the individual performance of each participant). Crucially, both participants are informed that this pool of shared profits is distributed equally between both interaction partners at the end of the task. If the outlook of shared profits is sufficient to produce retrieval of observational SR bindings, the pattern obtained in the present study for the “romantic partners” condition should replicate. Future research is therefore needed to address this issue.

Third, another concern relates to the fact that interaction partners in the “stranger” condition showed no SR retrieval at all. *Post hoc* power calculations (see “Materials and Methods” section) showed that the achieved power in the “stranger” condition was sufficient to detect an effect of at least medium size. We can therefore conclude that the absence of SR retrieval effects in the “stranger” condition does not stem from insufficient statistical power. Several explanations are possible. On the one hand, it is possible that working on a task with one’s own romantic partner goes along with closer monitoring of the interaction partner, compared with working with a stranger. Thus, observational SR bindings in the “stranger” condition might suffer from a lack of additional attentional processing, resulting in weaker SR bindings (Logan, 1988). However, if this was truly the case, one would also expect group-specific differences in the memory test for prime observers (i.e., higher error rates in the “stranger” condition). Notably, error rates in the memory test did not differ between groups. We can therefore conclude that prime observers in both interaction partner conditions attended to and consequently encoded observed prime responses to equal extent.

On the other hand, it is possible that the very fast overall RT level of the “stranger” condition affected retrieval of observational SR bindings. For instance, it is possible that the absence of a facilitation effect (on stimulus repetition compared with stimulus change probes) for compatible responses is due to the very fast overall RT pattern (i.e., a floor effect). In other words: Participants in this condition already responded so quickly that any further speed-up effect was negligible (or even impossible). However, if this line of reasoning is correct, one would expect that the interference effect (on stimulus repetition compared with stimulus change probes) for incompatible responses should in fact be stronger in the “strangers” compared with the “romantic partners” condition. That is because participants in the “romantic partners” condition are already so slow on a general level (i.e., reflecting a ceiling effect) that any further slowing due to retrieval of inappropriate responses has no further detrimental effect on probe performance. In our view, this is somewhat implausible, given that both effects, i.e., retrieval-induced facilitation for compatible responses and retrieval-induced interference for incompatible probe responses were more pronounced in the “romantic partners” condition. Nevertheless, we wanted to test this possibility empirically and performed quintile analyses.^6^ However, none of the effects of interest did interact with the quintile factor, indicating that overall differences in response speed cannot account for the observed pattern of results.

Related to the previous point, we want to emphasize that we cannot exclude that the overall speed differences between interaction partner conditions occurred as a *consequence* of (and hence was caused by) the manipulation. Put differently, working on the task together with one’s romantic partner might have relaxed participants to a certain degree due to this positive interdependency so that participants eased off (and also slowed down) a bit in their general wish to “get done” with the experiment. In turn, working with a stranger did not have this “easing” effect on participants, which is why participants in this condition responded significantly faster on a global level. Tentatively – although this is only a *post hoc* speculation – we want to point out that a similar main effect was also apparent in the study by Giesen et al. (2014, Exp 1). Namely, probe release RTs of participants in the cooperative condition were significantly slower than those of participants in the independent (Δ = 34 ms) or competitive (Δ = 30 ms) condition. Importantly though, the overall difference in global RT did not affect the retrieval of observationally acquired SR bindings, which was apparent in cooperative *and* competitive pairs (but absent in independent pairs). Against this background, we want to stress that the main effect of interaction partner condition cannot account for the qualitatively different pattern due to SR retrieval effects.

As another explanation to account for the absence of SR retrieval effects in the “stranger” condition, one might argue that opposite-sex strangers represent a potential threat for participants in a committed relationship. As a result, these participants might shield their relationship by (un-)consciously activating self-regulatory mechanisms that corrupt social interactions with interaction partners from the opposite sex (e.g., Koranyi and Rothermund, 2012). Findings by Karremans and Verwijmeren (2008) support this reasoning. They observed that imitation of an unacquainted, attractive opposite-sex interaction partner was reduced when participants were in a committed relationship, compared with singles. However, note that retrieval of observationally acquired SR bindings was also absent in the study by Giesen et al. (2014) when pairs of participants worked independently of each other and although about half of the pairings were same-sex interaction partners. In addition, probably as many participants of this sample were not involved in a romantic relationship and thus had nothing to shield against, but still did not show any effects of observational SR binding.

In our view, it makes more sense to regard the present absence of SR retrieval in the “stranger” condition as important replication of the null finding from the initial study by Giesen et al. (2014) when pairs worked independently of each other. According to Bandura (1986), not everything that is encoded through observation will also be retrieved later. With respect to the present paradigm, this means that one will not blindly incorporate any observational SR binding for one’s own action regulation. We therefore believe that the absence of observational SR retrieval represents the *default* in situations in which the interaction partner is *not* socially relevant either in the specific task/situation (e.g., when interaction partners work independently of each other) and/or in terms of more permanent forms of personal attachment (e.g., romantic partners, close friends, etc.).

### Theoretical Implications

An important question is how one can explain the modulating influence of social interdependence that is apparent not only for retrieval of observational SR bindings, but also for action co-representation effects. Building on earlier findings from Aron et al. (1991), several authors argued that the overlap between mental representations of self and other reflects a possible mediating process (e.g., Hommel et al., 2009; Giesen et al., 2014; Maister and Tsakiris, 2016). That is, as relationships become more interdependent or closer, the mental representations of self and other will be more closely interconnected. Following this line of reasoning, one is more likely to represent the response of another person like one’s own response if that other person is a s*ocially relevant other* (e.g., a person with whom one interacts in a cooperative or competitive way). Hence, interacting with socially relevant others makes it more likely (a) to co-represent actions of a co-actor (Ruys and Aarts, 2010; Iani et al., 2011), but also (b) to rely on observationally acquired SR bindings to regulate one’s own actions (Giesen et al., 2014). However, when another person is not socially relevant (e.g., when both participants work independently of each other), people are more likely to keep mental representations of self and other more distinct and separated from each other.

In this respect, the present study supports the notion that our cognitive system requires a minimum degree of connectedness between actor and observer in order to utilize observationally acquired SR bindings for one’s own action regulation. Connectedness in this respect can be conceptualized as the extent to which the co-actor is socially relevant in a given situation. Importantly, perceiving another person as socially relevant might be the product of situationally induced dependencies (i.e., instructions to cooperate with or compete against a co-actor), but might also result from more chronic forms of personal attachment (e.g., romantic relationship status) that “bridge the gap” between co-actors whenever situational dependencies are absent. However, it is an unresolved issue whether the present findings would also generalize to other forms of close relationships (e.g., close friends, family members, or lifelong arch-enemies) or are restricted to romantic relationships, which show not only overlap in cognitive representations of self and other, but also share body representations (see Maister and Tsakiris, 2016).

In addition, the present findings advocate overlap between mental representations of self and other as a potential underlying mechanism in producing retrieval effects of observational SR bindings even if the task does not explicitly require representing the other’s action. To bolster this claim, future research is needed to detect other conditions that also go along with closer or more distinct self-other representations. A worthwhile endeavor would be to explore manipulations that allow for a more direct test, for instance by experimentally inducing overlapping versus separate self-other representations.

## Author Contributions

CG developed research idea, study, design, organized data collection and analyses, as well as manuscript preparation. VL responsible for data recruitment and analyses, involved in manuscript preparation. KR involved in manuscript preparation. NK involved in development of research idea, and study design as well as manuscript preparation.

## Conflict of Interest Statement

The authors declare that the research was conducted in the absence of any commercial or financial relationships that could be construed as a potential conflict of interest.

## Footnotes

1 Twelve female participants in the “stranger” condition performed the task with a male confederate, since no male participant was available at the scheduled time of testing. Data of the confederate were excluded from all analyses.

2 Due to the small sample size of the “romantic partner” condition, outlier values may exert a stronger influence on small samples (compared with larger sample sizes), which makes it even more important to control for these biases (we thank an reviewer for pointing this out). Thus, we removed the couple with outlier value on relationship duration from the analyses. After excluding this couple from the sub-sample of all romantically involved interaction pairs (remaining *N* = 20), groups no longer differed in relationship duration (romantic partners: *M*_duration_ = 3.2; strangers: *M*_duration_ = 2.3), *t*(48) = -1.40, *p* = 0.16. Measures of relationship quality were unaffected by removal of the outlier couple and remained statistically equal between groups (romantic partners: *M*_RAS_ = 4.3; strangers: *M*_RAS_ = 4.3, |*t*| < 1). Most importantly, removal of the outlier couple did not affect ANOVA results: The three-way interaction of stimulus relation, response compatibility, and interaction partner remained significant, *F*(1,48) = 3.07, *p* = 0.043, $\eta_{p}^{2}$ = 0.06 (one-tailed; given our specific prediction with regard to the nature of the three-way interaction, a one-tailed test is allowed and recommended, see Maxwell and Delaney, 1990, p. 144). The Stimulus Relation × Response Compatibility interaction remained significant in the romantic partner condition, *F*(1,19) = 5.03, *p* = 0.037, $\eta_{p}^{2}$ = 0.21, compared to the stranger condition, *F* < 1. All results indicate that the Stimulus Relation × Response Compatibility × Interaction partner interaction was not due to the duration of the current relationship.

3 Probe release RTs below 250 ms or more than 1.5 interquartile ranges above the third quartile of the individual distribution of probe release RTs were regarded as outliers (Tukey, 1977).

4 The same 2 (stimulus relation) × 2 (probe response compatibility) × 2 (interaction partner) ANOVA on mean probe hit RTs as dependent measures yielded similar (though somewhat noisier) results. Specifically, the three-way interaction just missed conventional levels of significance with *F*(1,50) = 2.59, *p* = 0.057 (one-tailed), $\eta_{p}^{2}$ = 0.05. For illustrative purposes, we performed follow-up analyses similar to those reported in the main text to make sense of the underlying data pattern prevalent for probe hit RT. Accordingly, the Stimulus Relation × Response Compatibility interaction was significant for probe hit RT in the romantic partner condition, *F*(1,21) = 7.52, *p* = 0.012, $\eta_{p}^{2}$ = 0.26, but was completely absent in the stranger condition, *F* < 1. However, we refrain from interpreting results for probe hit RTs, since they are confounded with movement speed, and are hence no ideal performance indicator.

5 We thank an reviewer for drawing our attention to this important alternative explanation.

6 Specifically, we computed quintiles based on each participant’s individual probe release RT distribution. We then ran a 2 (stimulus relation) × 2 (response compatibility) × 2 (interaction partner) × 5 (quintile) mixed-models ANOVA. One participant had to be excluded from this analysis due to empty cells, meaning that data of *n* = 51 participants entered into the analysis. However, the quintile factor did not interact with the effects of interest: Specifically, neither the stimulus relation × response compatibility × quintile interaction, *F*(4, 46) < 1, *p* = 0.67, nor the four- way interaction of stimulus relation × response compatibility × interaction partner × quintile interaction, *F*(4,46) = 1.76, *p* = 0.15, reached significance.
